# BJS Academy website: *#LeadingSurgicalEducation*

**DOI:** 10.1093/bjsopen/zrac078

**Published:** 2022-05-09

**Authors:** Jonothan J. Earnshaw

**Affiliations:** BJS Academy Director @JJEarnshaw

**Figure:**
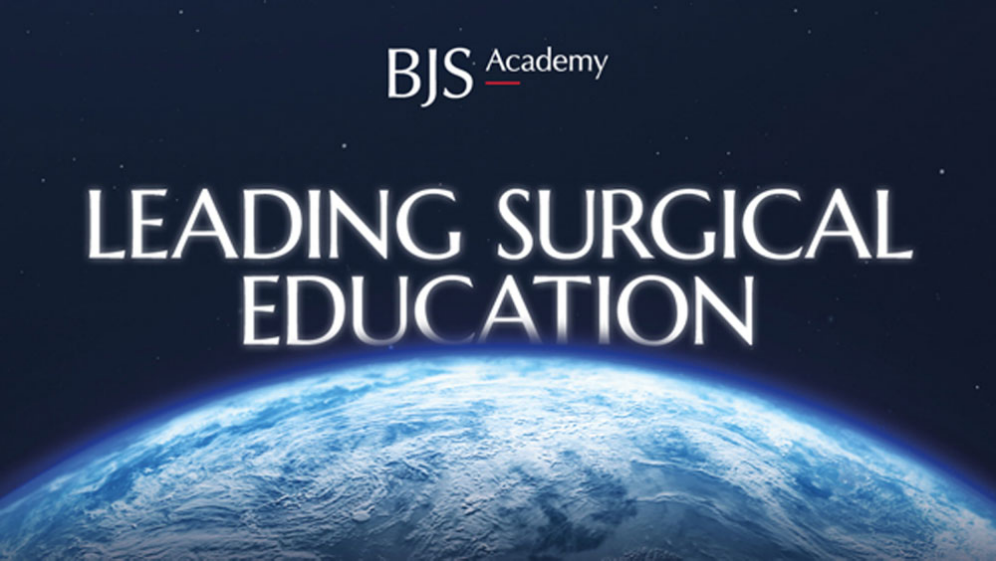


The BJS Society owns the two renowned surgical journals BJS and BJS Open. The Society considers its major responsibility to publish the best surgical research that promotes the advancement of surgery and surgical techniques. A secondary objective of the Society is to improve education in surgery. Formerly, this was done through organized lectures and workshops. The Covid pandemic has dramatically changed how much education is delivered, with a wholesale move to online provision. The BJS Academy is a new website that aims to become the leading forum for surgical education.

Many surgical websites have been developed; what is so different about the BJS Academy? The Academy is different in three ways. First, it is designed to add value to BJS and BJS Open. Second, the Academy will produce educational material with the BJS brand of quality that will appeal to all surgeons, but with an additional focus on surgeons in training through the Young BJS section. Finally, and perhaps most importantly, the Academy aims to be inclusive of surgeons worldwide, and will contain content for surgeons in low- and middle-income countries.

The Academy will also offer certified online academic courses in surgical research through the University of Edinburgh: the BJS Institute. In time, other Universities worldwide may take advantage of a similar partnership.

Most of the material on the Academy website will be free to access, though there will be a password-protected area for subscribers and surgeons belonging to partner organizations. In the open access part there will be cutting edge blogs to highlight material from BJS Society journals, continuing education for trained surgeons, digests of randomized clinical trials, surgical science and social media, and regular focussed updates on surgical topics of current interest.

Please visit the website at www.bjsacademy.com, and join the Academy. Let us know what you think and help us build a website that surgeons worldwide will turn to for information and education.

